# A Smart, Cost-Effective Programmable Gas Flowmeter Retrofitted from a Glass Rotameter

**DOI:** 10.3390/s26134020

**Published:** 2026-06-24

**Authors:** Xingcai Qin, Qi Cao, Zhiyuan Yuan, Yifan Hao, Sen Liu, Lianhui Wang

**Affiliations:** Key Laboratory for Organic Electronics and Information Displays, Jiangsu Key Laboratory for Biosensors, Institute of Advanced Materials (IAM), Nanjing University of Posts and Telecommunications, Nanjing 210023, China; 1023061702@njupt.edu.cn (Q.C.); 17537949055@163.com (Z.Y.); 1224066627@njupt.edu.cn (Y.H.); 1023061616@njupt.edu.cn (S.L.)

**Keywords:** programmable flowmeter, glass rotameter, computer vision, MATLAB–Arduino communication, stepper motor-actuated valve

## Abstract

Programmable gas flowmeters with remote reading and control are increasingly needed in the era of automation and AI. However, commercially available options remain expensive, hindering their adoption. We therefore developed a cost-effective programmable flowmeter as a compact device based on a low-cost glass rotameter. This system consists of a reading unit (webcam + rotameter), a control unit (development board + stepper motor-actuated valve) using an open-loop pre-calibrated step-to-flow matrix, and a terminal interface. The side-view imaging of the glass rotameter avoids occlusion of the rotor by the scale, enabling reliable rotor identification using well-established algorithms. The flow rate is derived by mapping the normalized rotor position to the scale instead of recognizing the scale markings or numerals. Two terminal configurations are offered: USB-connected (PC + MATLAB) and embedded (Raspberry Pi + Python). The USB-connected configuration uses direct serial communication between MATLAB and Arduino for stepper motor step control, ensuring fast response and good compatibility. The prototype retains manual control and direct eye reading. Experiments demonstrated strong programmability, fast response (0.2 s), high accuracy (mean error < 3%), and stable reading (fluctuation < 0.5%). This cost-effective yet programmable gas flowmeter is expected to benefit gas sensor development and accelerate automation in fluid-related fields.

## 1. Introduction

Gas flowmeters [1,2] are fundamental sensors/instruments widely used in numerous fields [3,4] such as industrial production [5], metrology, environmental protection, biomedical research, gas delivery, sensor development [6,7,8], scientific research, etc. Programmable gas flowmeters that offer capability of remote reading and control are increasingly required for a wide range of applications [9,10,11,12,13,14,15,16,17], especially in the era of automation and artificial intelligence (AI) [13,18]. However, commercially available options like MEMS mass flow sensors [3,10,19,20,21] remain expensive, limiting their applications and impeding the pace of automation.

In contrast, low-cost glass rotameters, by themselves, do not possess the capabilities of digital reading and remote control. Glass rotameters [22], which feature naked-eye reading (or direct reading) and manual control, have merits of low cost, simple but reliable structure, ease of operation, and long lifespan. However, their lack of digital reading and remote-control capabilities render them unsuitable for automated systems or restricted environments, making them seem obsolete in the modern era.

Computer vision (CV) provides a promising means to remotely read the flow rate of glass rotameter [23,24], while low-cost development boards enable programmable flow control. Their combination offers a path to low-cost programmability.

Previous attempts [23,24] at computer vision-based reading emphasized the development of a new image-recognition algorithm to read the flow rate like the human eye. Front-view imaging of the flowmeter was taken, and it was attempted to identify both the rotor and the scale in the same snapshot using a complicated algorithm. This method, however, faces significant challenges due to occlusion between the rotor and the scale. Consequently, it usually requires prominent rotors, controlled lighting conditions, and complex image processing algorithms, often at the expense of direct visual readability and measurement accuracy.

The booming development board ecosystem, with its wide array of accessories, facilitates bridging the electronic and physical worlds. A promising way for programmable control of flow rate is to use a stepper motor to actuate a conventional manual throttle valve [25,26,27]. Stepper motors are widely used in numerous applications requiring position control, like motorized linear stages. By rotating in precise steps or angles, a stepper motor in a stage could linearly move the mounted plate to a designated position. They have merits of simplicity, directness, high sensitivity, accuracy, repeatability, a long lifespan, etc. Throttle valves are a type of regulating valve, commonly used in glass rotameters, that control gas flow rate by manually rotating a knob or handle. Thus, stepper motor-actuated regulating valve could quickly and accurately adjust the gas flow rate. However, it remains challenging yet innovative to achieve an optimal combination of cost-effective development boards and compatible peripheral components, minimize overall learning burdens, implement communication and computation co-design, ensure seamless hardware–software integration, and maintain fast and reliable PC-to-development board communication.

Low-cost and compact development boards, such as Arduino, ESP32, and Raspberry Pi, are popular platforms for driving stepper motors. Arduino, an affordable and open-source electronics platform [28,29,30], could operate stepper motors and communicate with a host computer. Raspberry Pi [31,32], a cost-effective yet fully functional mini-PC, not only has GPIOs for direct stepper motor control but also possesses the capability to perform image processing as a terminal. Both the Arduino and Raspberry Pi are open-source, user-friendly, and equipped with a huge ecosystem that offers plenty of support, all of which would further reduce the R&D expenses. For example, edge computing combined with sensors and a stepper motor [6] shows potential for electronic nose systems, offering advantages over conventional designs.

To address those challenges and overcome these limitations, we hereby developed a novel programmable gas flowmeter as a compact physical device based on glass rotameters, which enables remote flow rate reading and control. This system uses computer vision for reading and a development board with a stepper motor-actuated valve for flow rate control, all within an integrated, compact structure. A pragmatic and cost-effective flow rate reading method was introduced. It uses side-view (rather than front-view) imaging of the rotameter, employs a well-established image-recognition algorithm to identify the rotor, and obtains the flow rate by mapping the rotor’s normalized position to the corresponding scale rather than recognizing the scale markings and numerals. Side-view imaging avoids occlusion of the rotor by the scale, which simplifies image recognition, enables the use of a well-established algorithm, and preserves the rotameter’s inherent direct eye-reading capability. The normalized-position method is simpler and more accurate than scale recognition. The developed prototype consists of three core components: a reading unit (a webcam and a glass rotameter), a control module (a development board and a stepper motor-actuated regulating valve), and a terminal interface. This terminal interface offers two configurations to suit different requirements: a USB-connected type (using a PC running a custom MATLAB app) and an embedded type (using a Raspberry Pi 4B running a self-developed Python app).

Additionally, the developed programmable flowmeter retains the inherent manual control and direct-reading capabilities of the original glass rotameter, offering both programmable and manual operations. By seamlessly merging the affordability and simplicity of glass rotameters with modern programmability, this work provides a practical solution to bridge the gap between the high cost of digital flowmeters and the growing demands of automation and AI (especially embodied AI).

## 2. Materials and Methods

### 2.1. Materials and Parts

Two different glass rotameters (GR), designated as GR1 and GR2 for subsequent reference, were employed. GR1 was an LZB-4WB model with a measurement range of 0–3 L/min, and GR2 was an LZB-3WB model with a range of 0–0.3 L/min. Manufactured by Senlod Co. Ltd. (Nanjing, China), both used a black ball as the rotor.

Two different stepper motors with their respective drivers were used, assigned as SM1 and SM2. SM1 was a Planet Gear Stepper Motor (model: 42BYGH40-18-22A-C5.18) along with its TB6600 driver from DFRobot (Zhiwei Robotics Corp., Shanghai, China), while SM2 was a universal 28BYJ-48 paired with a ULN2003 driver.

Two throttle valves (ThV1 and ThV2) were used for flow adjustment. ThV1 was DFlok SS-N31-4-A from Zhenjiang Defeiyiliu Valve Ltd. (Zhenjiang, China), and ThV2 was BYQD-09 from Shanghai BingYu Fluid Tech Ltd. (Shanghai, China). The original handles of these valves were detached, and each stem was connected with a stepper motor shaft via a jaw coupling (model CFC-30×30 from XD Coupling Co., Ltd., Shenzhen, China). Each valve was mounted on a linear sliding rail to ensure smooth movement when being rotated.

To control the flow rate, a stepper motor, a throttle valve, and a glass rotameter were integrated into a single unit. Different combinations of these components were expected to influence the control performance. For demonstration, two groups were assembled: Group 1 included GR1, SM1, and ThV1, while Group 2 consisted of GR2, SM2, and ThV2.

An Arduino UNO R3 equipped with Adafruit motor shield was also used to actuate the stepper motors. Two universal USB Webcams were used to monitor the glass rotameters. A Raspberry Pi 4B with a 7-inch display was employed.

The gas flow channel was established using Luer taper and MasterFlex tube (Cole-Parmer Instrument Company, Vernon Hills, IL, USA) to connect a mini air pump, a valve, and a glass rotameter. In all the tests of this article, clean air was pumped into the gas channel. The pump used was the KLP01-E KC from Kamoer Fluid Tech (Shanghai) Co. Ltd. (Shanghai, China), which had a maximum flow rate of 2.6 L/min (liter per minute or LPM).

All components were securely mounted on a PMMA plate at designated positions.

### 2.2. Software

MATLAB 2021b, with the Arduino Hardware Support Package, was used to develop MATLAB scripts and applications (apps).

Arduino IDE 2.3 and Mind+ were used for the Arduino programming.

Python scripts and applications (apps) were developed using PyCharm 2025.1.3 and Thonny 4.1.7 integrated development environments (IDEs) with Python 3.12 and the OpenCV library.

### 2.3. Flowmeter Design

Two types of programmable flowmeters were developed for different applications: a USB-connected type and an embedded type. The former must be connected to a terminal PC via USB during operation, while the latter used an embedded Raspberry Pi 4B as a standalone terminal. Both flowmeters consisted of three essential subsystems: flow rate reading, flow rate control, and a terminal. Specifically, the reading subsystem incorporated a glass rotameter and a webcam mounted laterally. This webcam provided real-time monitoring of the rotor, capturing time-sequenced images that were sent to the terminal for processing. The lateral placement of the webcam, which captured the side view of the rotor, proved highly advantageous. This arrangement dramatically simplified the identification and position of the rotor because it largely eliminated the visual overlap of scales. 

**Figure 1:**
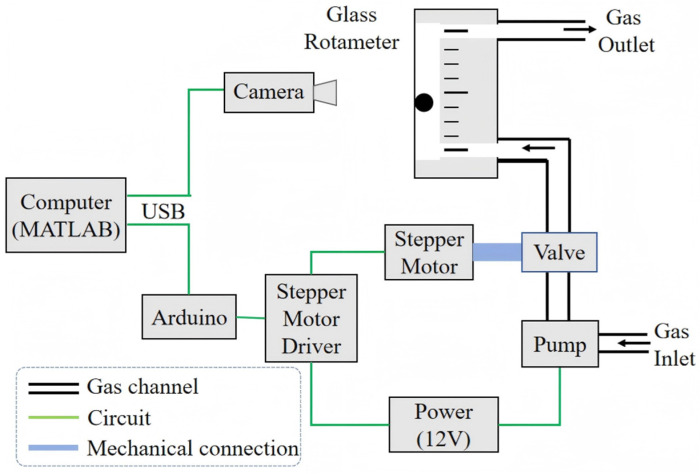
Schematic diagram of the USB-connected flowmeter. Key connections are color-coded: gas flow channel (double black line), electrical connection (single green line), and mechanical coupling between the stepper motor’s shaft and the valve’s stem (bold blue line). The flowmeter consists of 3 essential subsystems: flow rate reading, flow rate control, and a terminal. The reading subsystem integrates a webcam and a glass rotameter. The control subsystem incorporates a throttle valve, a stepper motor (with driver and 12 V DC power supply), and an Arduino Uno R3. The terminal is a PC running custom-developed MATLAB app. All components, except the PC terminal, are packaged into one compact device.

The schematic diagram of the USB-connected flowmeter is shown in Figure 1. The gas channel, circuit, and mechanical connection are clearly shown in different colors. 

**Figure 2:**
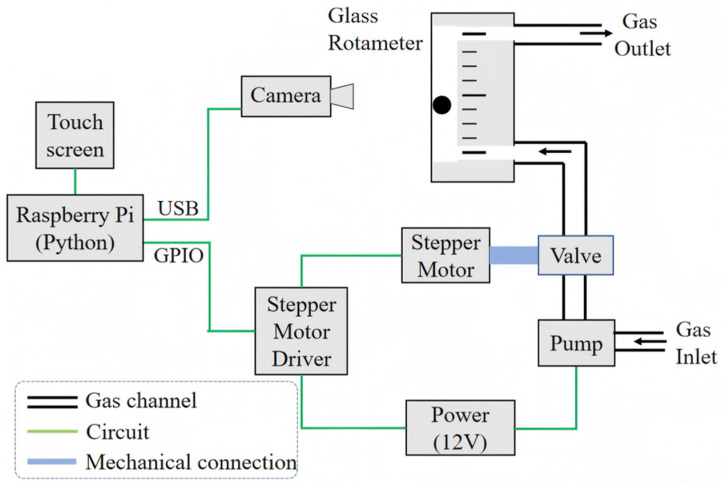
Schematic diagram of the embedded flowmeter. The key difference from the USB-connected design is the incorporation of a Raspberry Pi 4B executing a custom Python application, which supersedes the external PC and Arduino. Consequently, all components are consolidated into a single, compact, and standalone device, unlike the USB-connected version which relies on a PC terminal.

Figure 2 shows the schematic of the embedded flowmeter, which differs from the USB-connected type mainly by replacing the PC and Arduino with a Raspberry Pi. 

**Figure 3:**
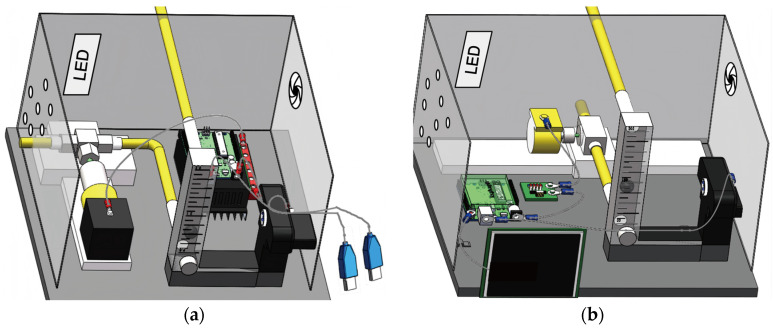
3D models of the flowmeters. (**a**) The USB-connected type; (**b**) the embedded type. To acquire a side-view snapshot, the webcam was positioned laterally to the glass rotameter. Yellow tubes represent the gas pathway.

Figure 3 shows the 3D models of the two flowmeters, with all components and their relative positions clearly indicated. As mentioned above, the webcam was laterally placed to capture a side view of the rotor, eliminating overlap of the scale marks. An LED was used for illumination, and a fan was added for heat dissipation. 

**Figure 4:**
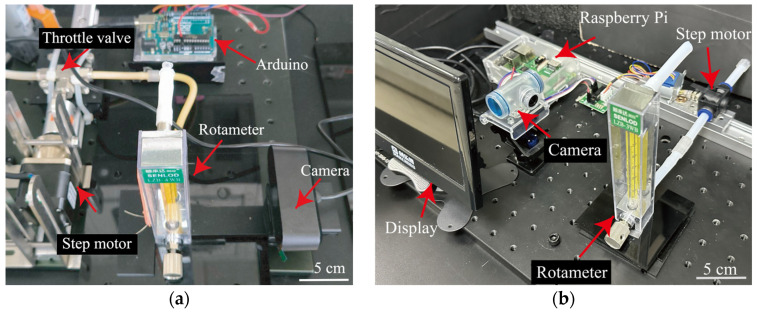
Photographs of the two flowmeter prototypes: (**a**) the USB-connected type; (**b**) the embedded type. The embedded version features a touch screen that also serves as a user interface for input and output.

The physical photographs of the two flowmeters are shown in Figure 4. All components are clearly shown and labelled. 

**Figure 5:**
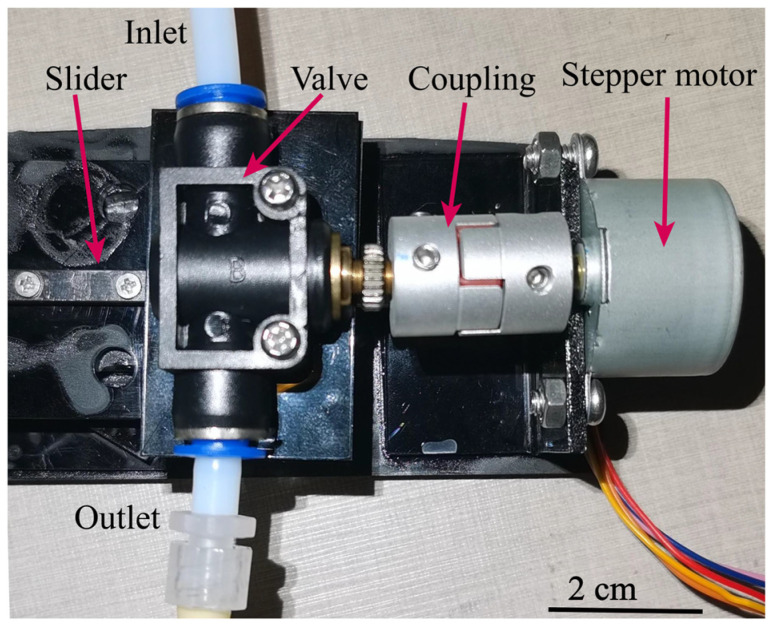
The connection between stepper motor and valve by a coupling (Group 2). The valve was mounted on a slider for free linear movement when the stepper motor rotated. The gas inlet and outlet are also shown.

The control subsystem incorporated a microcontroller (MCU-either Arduino or Raspberry Pi), a throttle valve, a stepper motor with its driver, and a jaw coupling that mechanically connected the motor shaft to the valve stem (Figure 5 and Figure S1). The valve was fixed on a sliding rail to ensure smooth movement during rotation. In addition to the independent throttle valve, there is also a built-in valve inside the glass rotameter to maintain the glass rotameter’s independence and allow for manual operation.

The terminal, which distinguishes the two flowmeters, was a PC with a custom MATLAB app for the USB-connected version and a Raspberry Pi 4B with a custom Python app for the embedded version. The PC terminal relied on an additional Arduino UNO R3 for motor control, whereas the Raspberry Pi terminal could drive the motor directly via its GPIO pins. Both terminals functioned by receiving user input, sending commands to adjust the flow valve, reading the flow rate via image analysis, and displaying the results.

The flowmeters also incorporated several subsidiary parts: a gas channel (Figure 1, Figure 2 and Figure 4) with a mini pump, throttle valve, glass rotameter, tubing and Luer tapers; electrical connections from the MCU GPIOs to the motor drivers (Figure S2); and a structural assembly where all components were fixed at designated positions on a PMMA plate. A custom cover (Figure S3) was also designed, exposing only the rotameter, display, and USB connector.

### 2.4. Method to Read the Flow Rate

**Figure 6:**
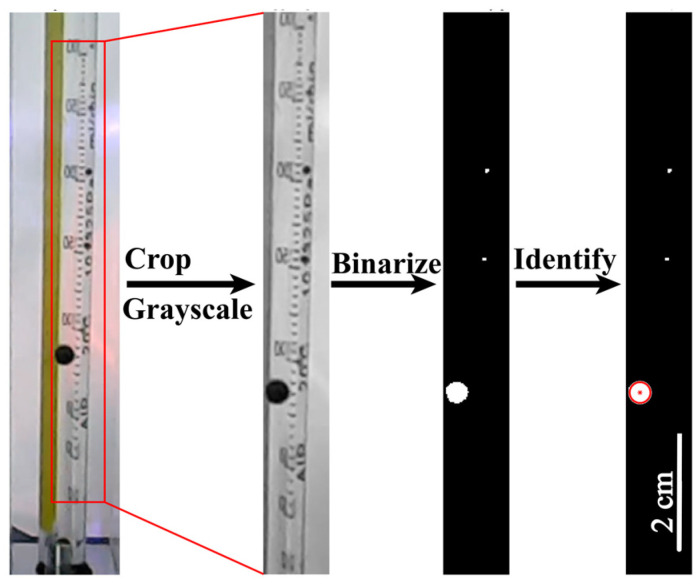
Image analysis procedure for rotor identification: original side-view snapshot of the glass rotameter, with the rotor (black ball) visible inside the tube; cropped and grayscale-converted image of the region between the highest and lowest scale markings; binarized image; rotor identification and center (the red circle and dot) calculation. The normalized position of the rotor was calculated using Equation (1). With side-view imaging, the rotor avoids occlusion by the scale markings and numerical readings—the markings appear beside the rotor, and the blurred numerals lie behind it. Thus, the scale does not hinder rotor identification—a key advantage over front-view approaches.

A pragmatic flow rate reading method is introduced, including two steps. First, side-view imaging of the rotameter is used to identify the rotor and its position; the flow rate is then derived from the normalized rotor position. Both flowmeters employ the same side-view imaging and a similar image-recognition algorithm to read the flow rate, as illustrated in Figure 6. A representative side-view snapshot of the glass rotameter is shown in Figure 6, where the rotor is clearly visible within the glass tube without occlusion by the scale: the markings appear beside the rotor, and the blurred numerals lie behind it. Thus, the scale does not hinder rotor identification—a key advantage over front-view approaches. 

As shown in Figure 6, the image processing algorithm comprised the following sequential steps: 1. Cropping the image to the area between the bottom and top scale markings of the glass tube. 2. Converting the cropped image to grayscale and subsequently binarizing it. 3. Identifying and localizing the rotor via circle detection and simultaneously acquiring its center coordinates. The detailed parameters for the circle detection are shown in S1. 4. Obtaining the normalized rotor position (δ) by Equation (1):

(1) δ=1−y/h

where, *y* is the vertical coordinate of the rotor’s center, and *h* is the height of the cropped image, which is also the distance between the highest and the lowest scale marks in the snapshot. For example, as shown in Figure 6, the Y-coordinate of the rotor center is 282.46 pixels (the origin at the top-left corner). The total height of the cropped image is 417 pixels. Consequently, the rotor’s normalized position was calculated as 0.323.

Then, the flow rate can be obtained by mapping δ to the corresponding scale value using a pre-established lookup table based on the rotameter scale. If the flow rate is linearly proportional to the rotor position, it can be obtained directly from Equation (2):

(2) $$Q=Q_{min}+\delta \cdot (Q_{max}-Q_{min})\;$$

where *Q* is the flow rate, *Q_min_* and *Q_max_* are the scale values at the lowest and highest flow rates, respectively, and the δ is the normalized rotor position.

This method offered a reading sensitivity that could potentially exceed that of direct visual reading of the glass rotameter. During operation, the webcam monitored the rotameter in real-time at a frame rate of 5 fps, and the flow rate was determined spontaneously through the aforementioned image analysis procedure. The complete cycle of image acquisition, analysis, and flow rate adjustment typically required approximately 0.1 s, resulting in a system response time of ~0.2 s.

The side-view imaging configuration significantly simplified image processing and was compatible with nearly all types of rotors. Additionally, positioning the webcam alongside the rotameter, rather than in front of it, preserved direct visual readability. Coupled with the use of a separate throttle valve, this design maintained the functional independence of a glass rotameter.

The ability for direct eye reading also makes it easier to notice any deviation between the actual flow rate and that read by computer vision. There are no significant differences or errors in this study. This high accuracy was achieved by using side-view snapshots of the rotor without occlusion, the well-established Hough circle detection algorithm, mapping the normalized rotor position to the scale via a high-resolution lookup table, and the fixed positions of the webcam and rotameter.

### 2.5. Method to Control the Flow Rate

Programmable flow rate control was achieved through an assembly comprised of a development board (Arduino Uno R3 or Raspberry Pi 4B), a compatible stepper motor with driver, and a throttle valve. As shown in Figure 5 and Figure S2, the shaft of the stepper motor was connected to the valve stem via a jaw coupling. The valve was mounted on a linear slider to accommodate its axial displacement during rotation, ensuring smooth and unrestricted movement. The development board governed the stepper motor programmatically, which precisely adjusted the valve’s opening to regulate the flow rate.

Side-view imaging and the additional throttle valve preserve the glass rotameter’s inherent manual control and eye-reading functions. As shown in Figure 3 and Figure S3, the knob and the scales of the rotameter remained accessible through the enclosure, enabling dual operation modes: programmatic and manual. This design effectively integrates the merits of both traditional glass rotameters and modern programmable flowmeters. The dual-mode flowmeter uses either manual or automatic mode, but not both. The two valves are in series. When one mode is active, its valve controls flow while the other stays 100% open. After each operation, the active valve returns to 100% open before mode switching.

### 2.6. Custom-Developed APP

**Figure 7:**
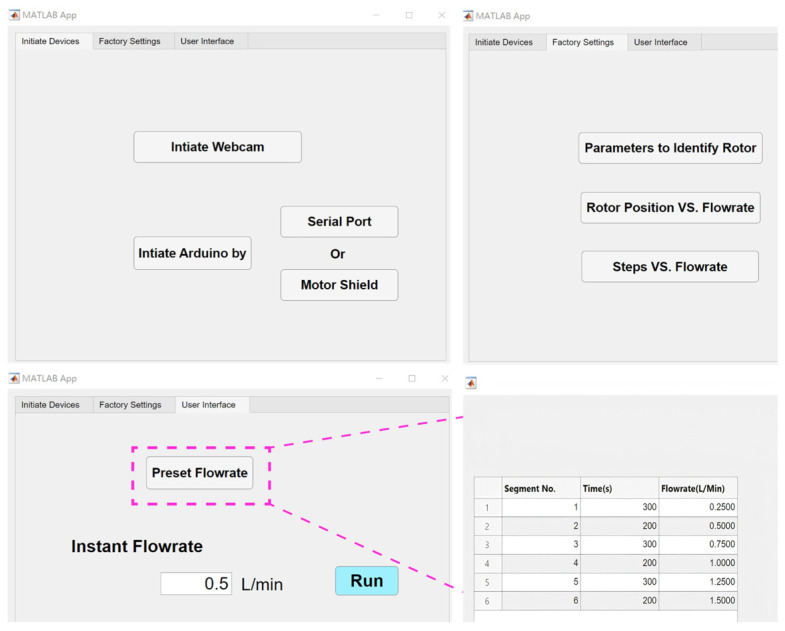
The graphical user interface (GUI) of the flowmeter’s terminal app (MATLAB). There are 4 Tabs: Tab of the device initiation, which is to establish connection between hardware (webcam and Arduino board) and host PC; tab of the factory setting for system calibration, which involves determining critical parameters such as the rotor identification model and the calibration matrices that correlate rotor position to flow rate and motor steps to flow rate; tab of the main operational interface, displaying the real-time flow rate; and defining a time-dependent series of flow rate setpoints by pressing the “Preset Flow rate” button. Once the setpoints are configured, pressing the “Run” button initiates the automated flow control sequence. The applications developed in Python and MATLAB share similar interfaces and core functionalities.

Taking the USB-connected version as an example, the custom-developed app features a tab-based interface, as shown in Figure 7 (there are four tabs in the app). The graphical user interface (GUI) includes the following key components. The “Initiate Devices” tab is to initialize hardware components (the webcam and Arduino Uno R3) and establish their connection with the host PC. The “Factory Setting” tab is employed for system calibration, which involves critical parameters such as rotor identification and the calibration matrices that correlate rotor position to flow rate and motor steps to flow rate. The “Parameters to Identify Rotor” and relationship between “Rotor and Flow rate” depend only on the rotameter. The “User Interface” tab serves as the main operational interface, which displays the real-time flow rate to the user. The “Preset Flow rate” button within the main interface allows users to define a time-dependent series of flow rate setpoints. Once the setpoints are configured, pressing the “Run” button initiates the automated flow rate sequence.

The applications developed in Python (Figure S4) and MATLAB share similar interfaces and functionality. Furthermore, owing to the cross-platform nature of Python, the Python application developed for the embedded version can be deployed on a PC-based terminal with minor modifications.

This study evaluated two primary communication methods between MATLAB and an Arduino board to drive the motor for flow rate control. The first method is serial communication, where a command including rotation direction and steps was transmitted from MATLAB to the Arduino. The Arduino then independently interprets and executes these commands. This method is characterized by its simplicity, universality, high compatibility, and rapid response, as it requires no additional hardware. Its functionality is independent of specific MATLAB or Arduino versions, making it a highly robust solution. The second method utilized the MATLAB Hardware Support Package, where the MATLAB code can control the stepper motor directly, but it requires the Arduino board to be compatible with the MATLAB version and necessitates an additional Adafruit Motor Shield to control the stepper motor effectively. For instance, the latest Arduino Uno R4 is incompatible with MATLAB 2021b and can only operate using the first method, whereas the Uno R3 is compatible with both. While functional, this method exhibits a slower response time than serial communication. Furthermore, without the motor shield, communication, though possible, becomes prohibitively slow for real-time flow rate control.

## 3. Results

**Figure 8:**
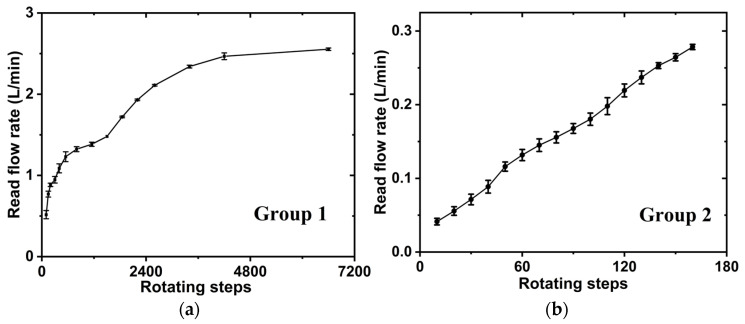
Calibration curves between the read flow rate and the rotating steps of the stepper motor. (**a**) For Group 1 tested in the USB-connected type; (**b**) for Group 2 tested in the embedded type. The components in each group incorporated a stepper motor with its driver, a throttle valve, and a glass rotameter. The specific components were mentioned in Section 2. The calibration curves depended on all the three components and the maximum flow rate of the gas delivery system (pump in this paper) and hardly on the control platform (USB-connected or embedded).

Upon launching the APP, the programmable flowmeter executes two initial procedures automatically: device initialization and factory settings (Figure 7). These steps establish hardware–software communication, and automatically set a half range flow rate and that verifies the reading. If a mismatch (>5%) is detected, it automatically re-calibrates and updates the calibration matrix, which maps the stepper motor steps to the corresponding flow rate readings (Figure 8). After these initialization steps, the device is ready for use, and the operational workflow then proceeds as follows:

1. User Input: The user opens the “User Interface” Tab, inputs the preset flow rate values and their corresponding durations, and initiates the process by clicking the “Run” button.
2. Command Generation: The APP calculates the required stepper motor rotation steps based on the preset flow rate and the pre-established calibration curve (Figure 8).
3. Actuation: Depending on the flowmeter type, the application either directly commands the stepper motor to rotate (embedded type) or sends a command containing the step count and direction to the Arduino Uno R3 for execution (USB-connected type).
4. Flow Reading and Feedback: The webcam monitors the glass rotameter in real-time, streaming snapshots to the terminal. Concurrently, the terminal analyzes each image to identify the rotor, calculates its normalized position, and determines the instantaneous flow rate by referencing a pre-stored position-scale matrix (“Rotor Position vs. Flow rate” in Figure 7). The resulting flow rate is then displayed on the application’s user interface in real-time (Figure 7).

The calibration curve, depicting the relationship between the flow rate of the glass rotameter and the rotation steps of the stepper motor, was experimentally determined and stored within the custom-developed APP as illustrated in Figure 8. The curve serves as the fundamental dataset (matrix) for the developed flowmeter. The shape of the calibration curve depends mainly on the specific combination of the stepper motor, throttle valve, and glass rotameter, and hardly on the control platform (USB or embedded). As previously noted in Section 2, the components were divided into two distinct groups (Group 1 and Group 2), and their respective calibration curves are presented in Figure 8.

The gas delivery system (e.g., pump or gas cylinder), particularly its flow rate and pressure, also influences the calibration curve. Therefore, the calibration curve needs to be retested when the gas supply changes, which is usually performed in the device initiation—the gas supply should be stable in the total process. As shown in Figure 8a, a delivery flow rate comparable to the rotameter’s maximum reading yields a gradual calibration curve (steep at low flow rates and gentle at high flow rates). In contrast, a delivery flow rate significantly exceeding the rotameter’s maximum capacity results in a more linear relationship, as seen in Figure 8b. The use of a proportional valve can further enhance this linearity.

**Figure 9:**
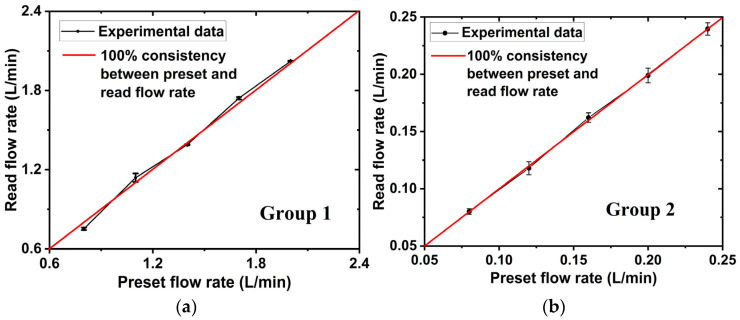
Consistency between the preset and read (actual) flow rates: (**a**) for Group 1 tested in USB-connected type; (**b**) for Group 2 tested in embedded type. The red lines indicate ideal 100% consistency between preset and read flow rate, while black dots with error bars represent experimental data. Both groups exhibit good consistency with mean absolute relative error less than 3% across the tested range (mean error < 3%).

Figure 9 demonstrates the close agreement between read (actual) and preset flow rates for both flowmeters. In the figure, red lines indicate ideal 100% consistency, while black dots with error bars represents experimental results. As we stated earlier, Group 1 and Group 2 differ in hardware components (throttle valve, stepper motor, and glass rotameter). The calibration results and errors depend primarily on these component differences, and hardly on the control platform (USB vs. embedded). Therefore, we label the panels by Group (1 or 2) rather than by platform. In this paper, Group 1 was tested only in the USB-connected type, while Group 2 was tested only in the embedded type. The excellent agreement further verifies the precision of the flow-control and the flow-reading methods. As configured, the USB-connected flowmeter utilized the glass rotameter, stepper motor, and throttle valve from Group 1, while the embedded flowmeter utilized those from Group 2. A significant deviation between the preset and actual flow rates would necessitate re-calibration of the system.

**Figure 10:**
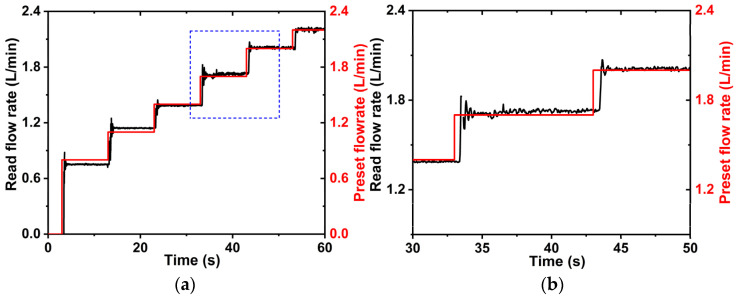
(**a**) Validation of the programmable flowmeter (Group 1); (**b**) zoomed-in view of (a), showing the response time and delay between the preset and read flow rates. The red line represents preset flow rates over time, whereas the black line indicates actual flow rates. The results demonstrated that the developed flowmeter exhibited excellent programmability, good consistency between set flow rate and actual flow rate (mean error < 3%), fast response (typical value of 0.2 s, <0.5 s), stable readings (fluctuation < ±0.5%), and good repeatability, indicating its excellent performance. The response time was measured from the start of flow change to 95% of the stabilized flow rate, as is the case for most sensors.

Programmability of the developed flowmeter is demonstrated in Figure 10, which shows that the actual flow rates accurately track the time-variant preset values. The results confirm that the system provides a real-time, stable, and precise flow control response. Overall, the flowmeter exhibits excellent performance, characterized by its fast response (typical value of 0.2 s, maximum 0.5 s), high accuracy (mean error < 3%), stable reading (fluctuation < 0.5%), good repeatability, ease of use, and strong programmability. Calculated from the data in Figure 9 and Figure 10, the mean absolute relative error and standard deviation for Group 1 are 0.78% and 1.1%, respectively, and for Group 2, they are 2.77% and 3.75%, respectively. Thus, the mean absolute relative error is below 3% across the tested range (mean error < 3%). The system also performs an auto-check during warm-up and at each setpoint change, triggering an auto-recalibration if the deviation exceeds 5%, thereby ensuring consistency and repeatability. Furthermore, during 1 h of continuous operation (typical for our use), the 0.2 s response time was consistently maintained, and the CPU utilization remained stable without an upward trend. This verifies the underlying flow-reading and control method as an effective, accurate, and versatile solution.

Clearly, this programmable flowmeter uses an open-loop control system based on pre-calibrated step-to-flow matrix. It offers fast response but requires a stable gas supply. Therefore, an automatic check during device initialization is necessary. An unstable gas supply may cause a significant deviation between the preset and actual flow rates. Mimicking human hand–eye coordination, an immediate closed-loop control system is envisioned as a future improvement. Such a system would supply more high consistency between the actual and preset flow rate, would not require pre-defined calibration curves, and would be independent of the delivery system characteristics, which is the focus of our next work.

## 4. Discussion

For the flow rate reading method, side-view imaging of the rotameter avoids occlusion of the rotor by the scale. This simplifies rotor identification, enhances identification accuracy, enables the use of well-established algorithms, and preserves the rotameter’s inherent functions of manual control and direct eye reading. Deriving the flow rate from the normalized rotor position—whether by direct calculation (linear case) or by a pre-established lookup table (nonlinear case)—simplifies the reading process and improves the accuracy. In contrast, previous research based on front-view imaging attempted to identify both the rotor and the scale simultaneously using complex algorithms. That work focused on developing advanced image-recognition algorithms to read the rotameter like the human eye, whereas the present work focuses on developing a pragmatic, programmable flowmeter in a simple manner, preferring well-established image-recognition algorithms.

The communication methods between MATLAB and Arduino for driving a stepper motor were carefully examined. Direct serial communication is recommended, primarily for its rapid response and good compatibility, as well as for its simplicity and cost-effectiveness. In this way, MATLAB derives the required motor steps and duration from the preset value and pre-calibrated matrix, sending them to Arduino in one command, after which both devices execute their code separately. Communication based on the MATLAB Support Package for Arduino Hardware and an additional Adafruit motor shield (for controlling a stepper motor) can also meet the requirements, albeit with a slightly slower response. Communication relying merely on the Arduino support package is not acceptable due to its slow response—in fact, it is too slow because each step of the stepper motor needs one command, taking ~0.3 s per step.

This programmable flowmeter uses an open-loop flow control system based on a pre-calibrated matrix that maps the stepper motor’s step count to the flow rate of the glass rotameter. Therefore, a verification step is performed during its warm-up period, and a stable gas supply is necessary; otherwise, the preset and actual flow rates may deviate significantly. It was designed for a system with stable supplies (e.g., pumps or gas cylinders), which are standard in laboratory settings. The actual flow rate increases or decreases with the upstream pressure (Figure S5). To ensure accuracy, we implemented three measures: (1) auto check during warm up with recalibration if deviation > 5%; (2) deviation check on each setpoint change with alert and requesting of auto-recalibration if the deviation exceeds 5%; (3) preserved eye reading for independent verification. Additionally, supply monitoring can indicate leakage or blockage. However, open-loop flow control systems typically exhibit faster response than closed-loop ones.

Furthermore, the former two measures can compensate for long-term wear, but if recalibration is requested frequently or repeatedly, it indicates a probable hardware issue (e.g., loosening or breakage), in which case the device should be returned for repair.

A circular rotor is preferable for the proposed method, but other shapes can be accommodated with minor code modifications. The clear outline of the float, enabled by side-view imaging (without scale overlay), allows easy adaptation to non-spherical floats by switching between top-edge detection and centroid computation. Thus, the inherent advantages of side-view imaging and normalized-rotor-position-based flow rate measurement remain intact. The developed device is suitable for measuring colorless gases or liquids.

Future work will focus on three improvements: incorporating multiple cameras to reduce the device size, adopting servo and proportional valves to enhance the accuracy, and implementing closed-loop control (e.g., PID) to simplify the operation and eliminate the effects of gas supply variations.

## 5. Conclusions

This study presented an all-in-one solution for a programmable flowmeter by integrating flow rate reading and control into a compact system with seamless hardware–software integration. Two versions—USB-connected (PC + MATLAB) and embedded (Raspberry Pi + Python)—were developed to suit different application scenarios. The proposed side-view imaging method and normalized rotor position-based reading effectively avoid scale occlusion, enabling reliable flow measurement using well-established algorithms. Direct serial communication between MATLAB and Arduino is recommended for driving a stepper motor due to its fast response and good compatibility. An open-loop control system based on a pre-calibrated matrix (mapping stepper motor steps to flow rate) provides fast response and sufficient accuracy for practical use. The resulting flowmeter is low-cost, retains manual control and direct eye reading, and offers real-time display.

The experimental results demonstrated excellent performance in terms of strong programmability, fast response (0.2 s typical, <0.5 s), high accuracy (mean error < 3%), stable reading (fluctuation < 0.5%), versatility, ease of use, and close agreement between the actual and preset flow rates. The device can operate in either programmable mode or manual control/direct-reading mode without sacrificing the merits of the traditional glass rotameter. The dual mode also makes it easy to notice any deviation between the actual flow rate (obtained by direct eye reading) and the flow rate read by computer vision.

The developed programmable flowmeter has potential applications in gas sensor development and industrial automation, aiming to accelerate automation in flow-related fields.

## Data Availability

The original contributions presented in this study are included in the article/Supplementary Materials. The code is available from the corresponding authors upon reasonable request.

## Supplementary Materials

The following supporting information can be downloaded at: https://www.mdpi.com/article/10.3390/s26134020/s1. Figure S1: a photograph of the control unit. Figure S2: a diagram of the electrical connection between the Arduino Uno R3 and stepper motor driver. Figure S3: a photograph of the fully assembled flowmeter with its cover. Figure S4: the custom Python app. Figure S5: Influence of upstream pressure variation on the measured flow rate. S1: Functions and parameters to identify rotor.

## Abbreviations

The following abbreviations are used in this manuscript:

CV	Computer Vision
GR	Glass Rotameter
SM	Stepper Motor
ThV	Throttle Valve
