# Supplementary material for: A Smart, Cost-Effective Programmable Gas Flowmeter Retrofitted from a Glass Rotameter
Source: Sensors (Basel). 2026 Jun 24;26(13):4020. doi: 10.3390/s26134020 (PMC13363873; doi:10.3390/s26134020)
Supplement: Supplementary file 1 [file sensors-26-04020-s001.zip › sensors-4328336-supplementary.pdf]

Supporting Information for

A Smart, Cost-Effective Programmable Gas Flowmeter Retrofitted from a Glass Rotameter

Xingcai Qin*, Qi Cao, Zhiyuan Yuan, Yifan Hao, Sen Liu, Lianhui Wang*

Key Laboratory for Organic Electronics and Information Displays & Jiangsu Key Laboratory for Biosensors, Institute of Advanced Materials (IAM), Nanjing University of Posts and Telecommunications, Nanjing, 210023, Jiangsu, P. R. China;

*Corresponding Author.

Email: iamxcqin@njupt.edu.cn

Figure S1: Photograph of the control unit (Group 1).

Figure S2: Diagram of the electrical connection between stepper motor, its driver and GPIOs of Arduino Uno R3.

Figure S3: Photograph of the fully assembled flowmeter with its covers.

Figure S4: The custom Python app for the embedded flowmeter.

Figure S5: Influence of upstream pressure variation on the measured flow rate.

S1: Functions and parameters to identify rotor.

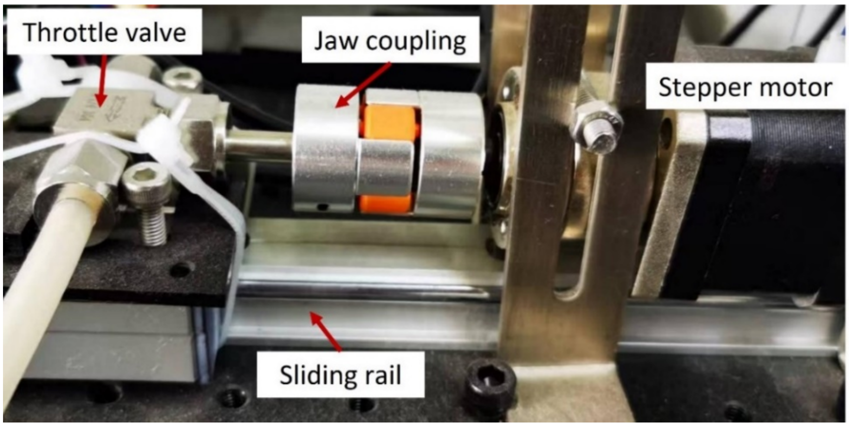

Figure S1. Photograph of the control unit and its mechanical coupling (Group 1). The unit comprises four key components: a throttle valve, a stepper motor, and a jaw coupling connecting the motor shaft to the valve stem, and a linear sliding rail. The valve is mounted on the rail, which enables its free linear travel during motor rotation.

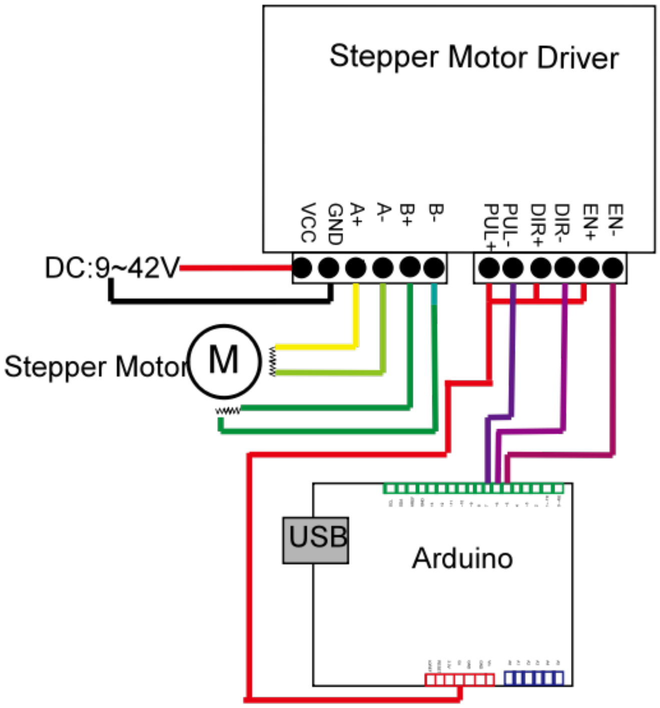

Figure S2. Diagram of the electrical connections between stepper motor, its driver and the Arduino GPIOs. The driver is powered by an external 12 V DC supply. The Arduino provides control signals via three digital I/O pins and a common ground (GND), connected to the corresponding inputs on the driver as illustrated.

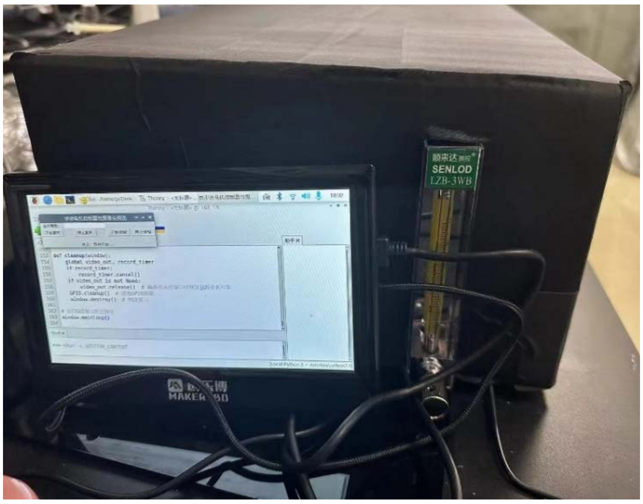

Figure S3: Photograph of the fully assembled flowmeter with its covers (the embedded version). Only the display and the glass rotameter are exposed outside the cover. The touchscreen is for interactive input/output, while the glass rotameter retains its functions of manual control and direct visual reading.

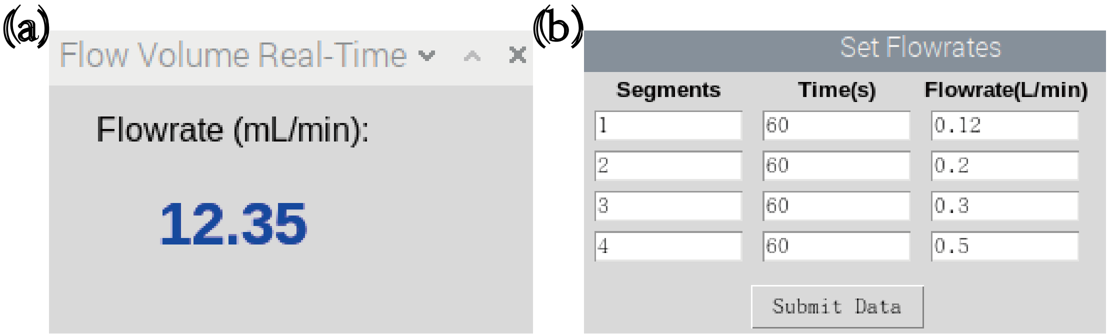

Figure S4. Custom Python app for the embedded flowmeter. (a) The main interface for real-time flow rate display. **(b)** The configuration interface that allows the user to preset flow rate setpoints and corresponding time durations.

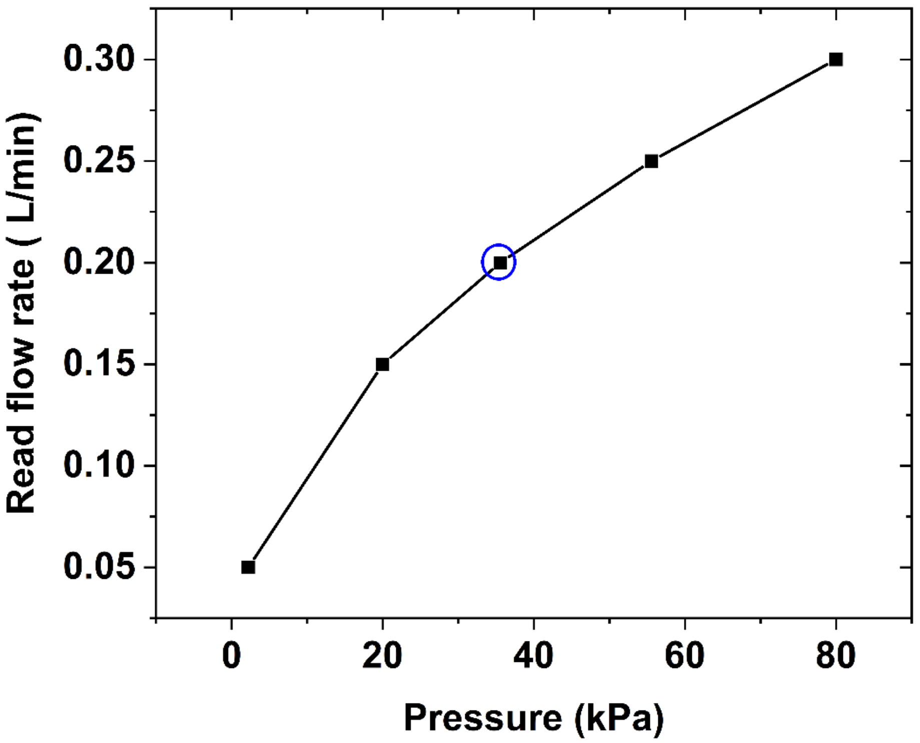

Figure S5. Influence of upstream pressure variation on the measured flow rate. The preset flow rate is 0.2 L/min (upstream pressure ≈35 kPa). The actual flow rate increases or decreases with the upstream pressure.

S1: Functions and parameters to identify rotor.

Preprocessing steps: as shown in Figure 6. It includes: Grayscale conversion → binarize. And then the built-in function with proper parameters can identify the circles.

MATLAB: *[centers,radii,metric]=imfindcircles(imgbwoa,[10,40],'Sensitivity',0.9);*

Python (OpenCV):
```
gray = cv2.cvtColor(roi_frame, cv2.COLOR_BGR2GRAY)
    blurred = cv2.GaussianBlur(gray, (9, 9), 2)
    circles = cv2.HoughCircles(blurred,
cv2.HOUGH_GRADIENT,dp=1,minDist=50,param1=50,
        param2=15,
        minRadius=5,
        maxRadius=18 )
```

These parameters were optimized for our specific camera setup and rotameter geometry.
